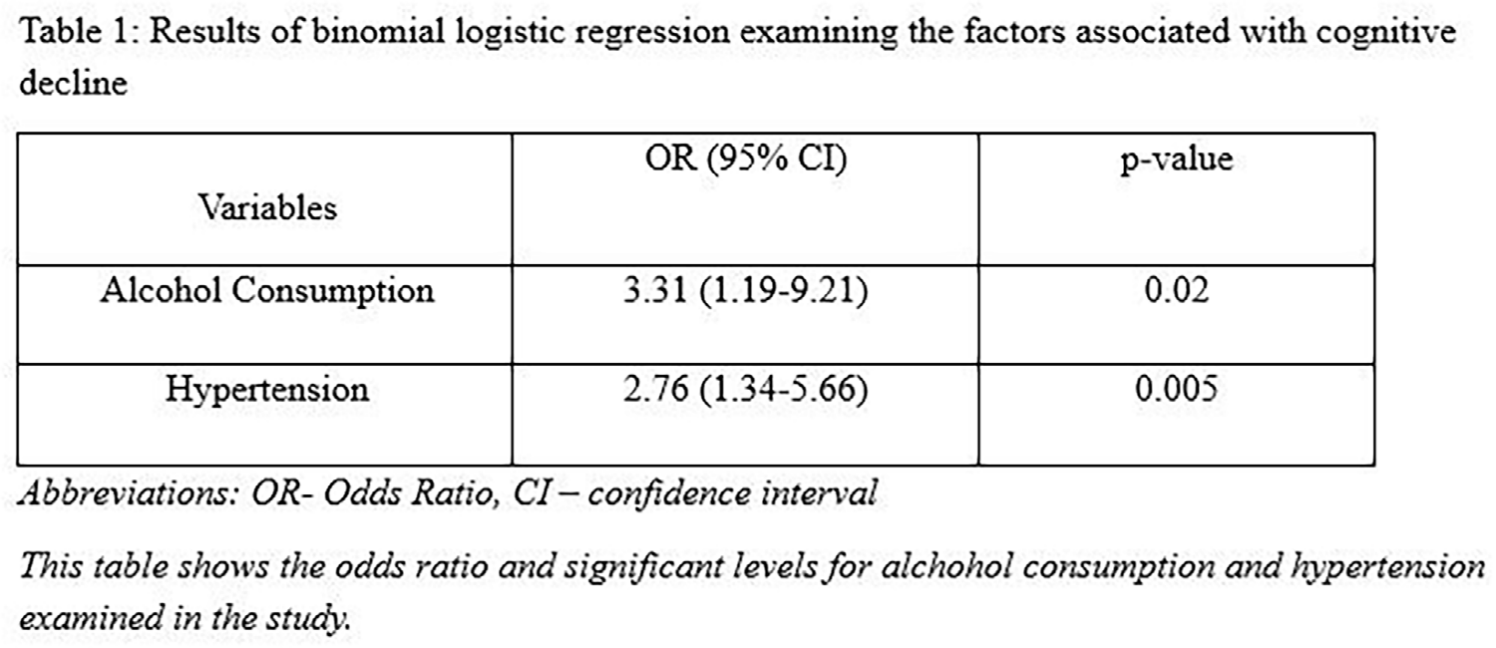# Exploring the factors impacting episodic memory decline in a longitudinal urban cohort

**DOI:** 10.1002/alz.092604

**Published:** 2025-01-03

**Authors:** Meghana R, Abhishek Mensegere Lingegodwa, Rajitha Narayanasamy, Vindhya Vishwanath, Meenakshi Menon, Divya N Mallikarjun, Amitha C M, Ajith Partha, Albert Stezin, Dev Kumar HS, Prathima Arvind, Sunitha HS, Goutham Velavarajan, Deepashri Agrawal, Jonas S. Sundarakumar, Thomas Gregor Issac

**Affiliations:** ^1^ Centre for Brain Research, Indian Institute of Science, Bangalore, Karnataka India

## Abstract

**Background:**

In the early stages of typical Alzheimer’s disease, there is a well‐documented pattern of memory deficits, especially episodic memory, substantiated with evidence of medial temporal lobe atrophy, specifically of the hippocampus in line with the memory deficits. Studies have shown that several other demographic, biological, and lifestyle factors influence memory and there is a need for identifying early risk factors and for the development of clinical intervention programs to delay or prevent cognitive decline. Therefore, the objective of this study is to explore the impact of various factors on episodic memory decline in an urban cohort.

**Method:**

A longitudinal analysis within the TATA Longitudinal Study of Aging (TLSA) examined episodic memory in a Bangalore urban cohort (n = 212). The analysis utilized data from baseline and a 2‐year follow‐up, employing the logical memory test for episodic memory assessment. Various demographic, clinical characteristics, and lifestyle factors were analyzed with episodic memory. Chi‐square and binominal logistic regression were used to analyze the data.

**Result:**

In a study sample of 212,51.4% women and 48.6% were men. The mean age of the study sample was 67.71 and the mean years of education was 14.9. There was a statistically significant association between alcohol consumption and episodic memory delayed recall score after two years [OR = 3.31 (1.19, 9.21)], indicating that people who consumed alcohol were 3.31 times more likely to experience a decline in episodic memory performance. Additionally, a significant association was identified between hypertension and delayed recall score [OR = 2.76 (1.34‐5.66)], implying that individuals with hypertension were 2.76 times more likely to exhibit episodic memory decline over the two years. Age, gender, education, smoking, diabetes, depression and anxiety were not significant with episodic memory.

**Conclusion:**

There was an association between alcohol consumption, hypertension, and episodic memory decline, indicating that individuals who engage in alcohol consumption or have hypertension are more prone to experiencing a decline in episodic memory over two years. This research study underscores the importance of Clinical programs promoting healthier lifestyles, particularly around alcohol consumption and blood pressure management, which could significantly mitigate memory‐related problems in aging populations.